# Rotational Atherectomy of Aorto-Ostial Calcification and Retained Native Leaflet to Facilitate Valve-in-Valve TAVI

**DOI:** 10.1016/j.jaccas.2025.103949

**Published:** 2025-05-10

**Authors:** Andrew R. Chapman, Lance Ng, Shaw Hua Kueh, Peter Barr, Jonathon White, Mark Webster

**Affiliations:** a Greenlane Cardiovascular Unit, Auckland City Hospital, Auckland, New Zealand; b Centre for Cardiovascular Science, University of Edinburgh, Edinburgh, Scotland; c University of Auckland, Auckland, New Zealand

**Keywords:** bioprosthetic aortic stenosis, rotational atherectomy, valve-in-valve TAVI

## Abstract

**Background:**

A 72-year-old patient presented with breathlessness and presyncope due to severe stenosis within a 25-mm Perimount bioprosthetic aortic valve. The patient had hepatic cirrhosis and was not a candidate for redo surgical valve replacement. A computed tomography transcatheter aortic valve implantation (TAVI) demonstrated severe aorto-ostial left main calcific disease, with dense calcification from the annulus to the inferior border of the left main ostium, which was thought to represent a retained native leaflet, with high risk of coronary obstruction.

**Case Summary:**

Using cerebral protection, rotational atherectomy was undertaken through the aorto-ostial calcification and retained leaflet, followed by left main stem percutaneous coronary intervention. A staged TAVI with a 23-mm Sapien S3 and a chimney stent for coronary protection was successful.

**Discussion:**

Although concomitant left main stem rotational atherectomy and TAVI have been described, no reports of rotablation to facilitate leaflet modification have been identified.

**Take-Home Message:**

Rotational atherectomy helped to modify the retained aortic valve leaflet and safely facilitate TAVI in a nonsurgical candidate.

## Visual Abstract

**Figure:**
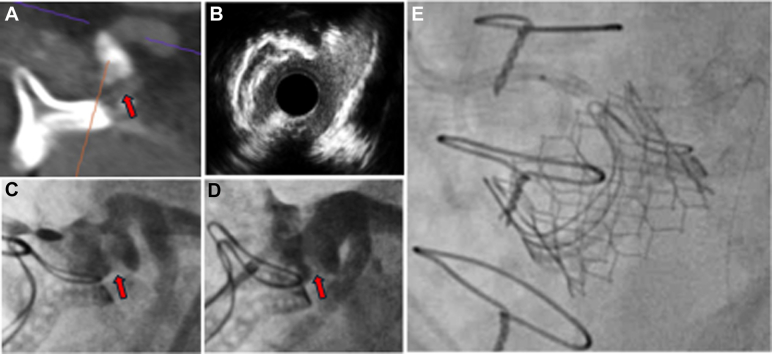

## History of Presentation

A 72-year-old man presented with increasing breathlessness and exertional presyncope. He had undergone surgical aortic valve replacement and a single saphenous vein bypass graft to the distal right coronary artery 6 years prior. The surgeon commented that the tricuspid aortic valve had severe calcification and severe aortic root calcification around both coronary ostia. Extensive annular decalcification was undertaken and a 25-mm Perimount valve (Edwards Lifesciences) was placed. After 6 years, the patient developed structural bioprosthetic valve degeneration. Transthoracic echocardiography demonstrated a mean transaortic valvular gradient which had increased from 11 to 41 mm Hg, with an aortic valve Vmax (maximum velocity across the aortic valve) of 4.1 m/s and dimensionless index of 0.3. There was mild aortic regurgitation through the valve and no significant paravalvular leak. The left ventricular systolic function was preserved with an ejection fraction of 55% and severe hypertrophy. There was mild right ventricular dilatation with mildly reduced systolic function. The symptoms and echocardiographic features were in keeping with severe bioprosthetic aortic valve stenosis due to structural valve degeneration (Valve Academic Research Consortium stage 3).

**Take-Home Message**

• Consider rotablation in patients with severe aorto-ostial calcification at high risk of coronary occlusion during TAVI.

## Past Medical History

The patient had a history of previously harmful alcohol intake but was now abstinent. There was evidence of liver cirrhosis on abdominal ultrasound, with features of portal hypertension including splenomegaly. Gastroscopy confirmed grade 1 varices with evidence of mild portal hypertensive gastropathy. Liver function was preserved (albumin: 32 g/L [range: 32-48 g/L], alanine aminotransferase: 27 U/L [<45 U/L], bilirubin: 28 umol/L [<25 μmol/L], alkaline phosphatase: 107 U/L [range: 40-130 U/L]). His liver disease was thought to be due to mixed alcohol-related and steatotic liver disease (Child-Pugh A score, Model for End-stage Liver Disease [MELD] score 10). Additional relevant medical history included hypertension, dyslipidemia, type 2 diabetes mellitus, and chronic pancytopenia thought to be related to chronic liver disease (Haemoglobin: 108 g/L [130-175 g/L], hematocrit: 0.335 L/L [0.400-0.520 L/L], platelet count: 43 (150-400), white blood cell count: 2.8 × 10^9^ [4.0-11.0], neutrophil count: 1.82 × 10^9^ [1.9-7.5], lymphocytes: 0.50 × 10^9^ [1.0-4.0]).

## Investigation

Invasive coronary angiography was undertaken as part of work up for valve-in-valve (ViV) transcatheter aortic valve implantation (TAVI). The right coronary artery graft was patent. It was difficult to engage the left main coronary artery with a range of standard diagnostic catheters, with a suggestion of a linear calcific obstruction in some views (Video 1).

**Figure 1:**
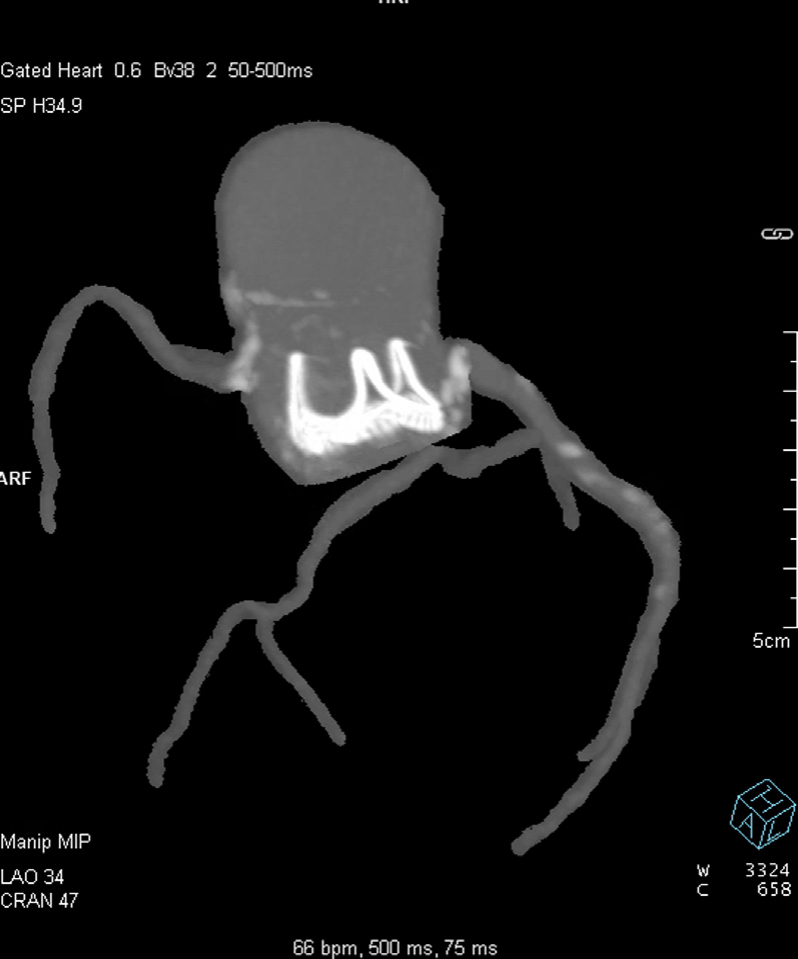
Transcatheter Aortic Valve Implantation Planning CT Scan Demonstrating Perimount Implant With Severe Calcification Abutting Left Main Stem

**Figure 2:**
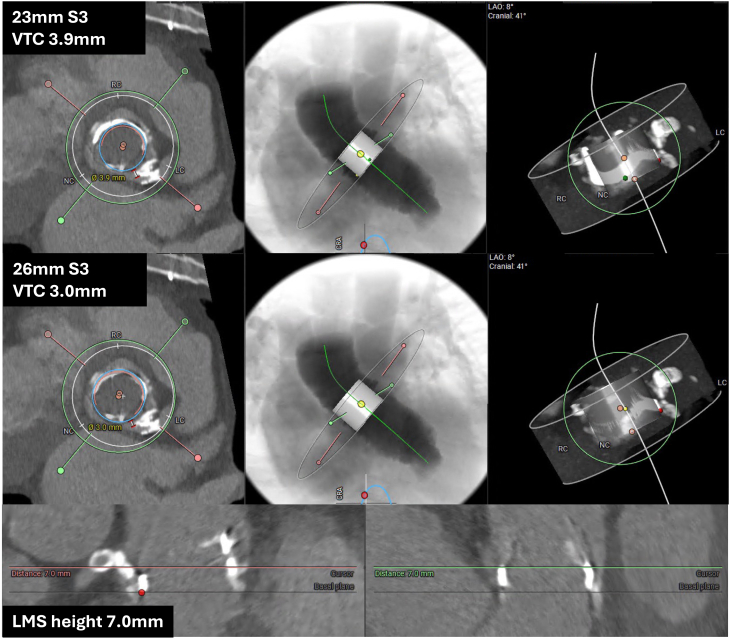
Virtual Valve-in-Valve Transcatheter Aortic Valve Implantation Planner LAO = left anterior oblique; LC = left coronary cusp; LMS = left main stem; NC = non coronary cusp; RC = right coronary cusp; VTC = virtual transcatheter valve to coronary ostium clearance.

A computed tomography TAVI scan demonstrated severe calcific disease at the left main coronary ostium extending superiorly to the sinotubular junction, with a bulky calcific structure extending from the annular plane to the inferior border of the left main ostium (Figure 1). The 25-mm bioprosthetic Perimount valve was in a normal position with severely calcified leaflets. The left and right coronary ostia heights were 7.0 mm. A 26-mm Sapien S3 (Edwards Lifesciences) is typically recommended for a 25-mm Perimount valve, but the virtual transcatheter valve to coronary ostium clearance (VTC) was just 3.0 mm. Using a 23-mm Sapien S3, the VTC increased to 3.9 mm (Figure 2). There was no significant iliofemoral disease.

**Figure 3:**
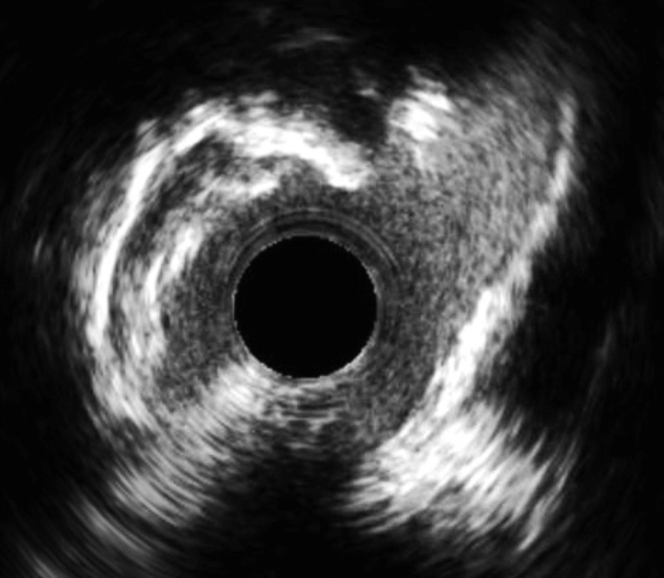
Intravascular Ultrasound of Left Main Stem Ostium

A repeat invasive coronary angiogram was undertaken with femoral access to facilitate further coronary assessment. It was difficult to engage the left main coronary artery, but this was eventually successful with an Amplatz left 1 guiding catheter (AL1) guide, a Balance, and a Whisper LS coronary wire. Intravascular ultrasound confirmed severe ostial left main coronary artery stenosis (minimal luminal area: 3.43 mm^2^) (Figure 3, Video 2). There was calcific disease in the body of the left main, and a further bulky linear calcific structure within the aortic sinus. Using an Abbott PressureWire X, a fractional flow reserve value of 0.77 was demonstrated in the healthy proximal circumflex.

## Management

This patient had severe symptomatic bioprosthetic aortic valve stenosis and obstructive left main stem disease, with a significant contribution from aorto-ostial calcification and a retained native aortic valve leaflet. The escalating symptoms of presyncope were a clinical concern. After multidisciplinary team assessment, it was thought his comorbidities precluded redo aortic valve surgery and he should be considered for percutaneous treatment.

The combination of a borderline valve to coronary clearance and bulky aorto-ostial calcification raised concerns that ViV TAVI carried a major risk of left main occlusion. A Basilica approach was not favored given the native leaflet was jailed behind the Perimount frame, the leaflets were thickened, and there was a significant contribution of aorto-ostial calcification. We therefore decided to undertake rotational atherectomy of the native leaflet and aorto-ostial calcification to facilitate stenting. The patient was fully appraised of the risks of major complication including annular perforation, myocardial infarction, embolic stroke, or death.

**Figure 4:**
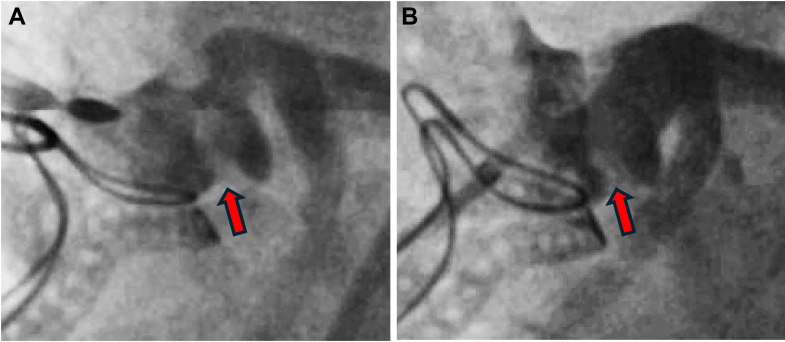
Before and After Rotablation Retained native leaflet calcification (arrow) and aorto-ostial calcification before (A) and after (B) modification with rotablation. Equipment list provided in Table 1.

A Sentinel Cerebral Protection system (Boston Scientific) was placed via the right radial artery to minimize the risk of cerebral embolization during atherectomy. Using a disengaged 8-F AL1 guide and a Rotafloppy wire in the distal left anterior descending, several 2.0 RotaPro burr passes were undertaken from the aortic root into the left main stem. A repeat intravascular ultrasound demonstrated significant atherectomy and calcium modification (Figure 4, Video 3). After a 5.0 Non-compliant (NC) balloon inflation, a 5.0 × 12 Megatron drug-eluting stent was then deployed (Video 4). We attempted to cover the ostium but minimize stent protrusion into the aorta because a chimney stent bailout was still considered likely during TAVI; however, during deployment, the stent interacted with ostial calcification and moved into the vessel. The stent was post dilated with a 6.0 × 12 NC at nominal pressure.

**Table 1:** Equipment List

Imaging • Preprocedural CT TAVI (Syngo Via software) and echocardiography • Opticross HD Intravascular Ultrasound (Boston Scientific) • Ultrasound machine (Philips Healthcare)
Access • Micropuncture needle and wire • .035 J wire and 8-F sheath • 3 × Proglide (Abbott Vascular) • Confida Brecker Guidewire (Medtronic) • 14-F eSheath (Edwards Lifesciences LLC) • 1 Hemostat
Coronary rotational atherectomy and stent • 8-F AL1 guide • 2.0-mm RotaPro Burr (Boston Scientific) • Sion blue (Asahi Intecc Medical) • Megatron 5.0 × 12 mm and 5.0 × 20 mm DES with 6.0 × 20 mm NC TREK
TAVI • 6-F AL1 guide • 5-F Pigtail catheter • 23-mm Sapien S3 (Edwards Lifesciences LLC)

AL1 = Amplatz left 1 guiding catheter; CT = computed tomography; DES = drug-eluting stent; TAVI = transcatheter aortic valve implantation.

**Figure 5:**
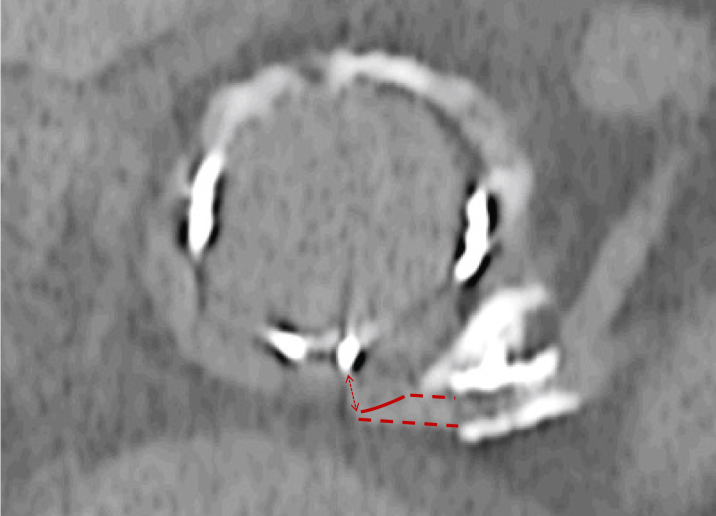
Computed Tomography Reconstruction Post Percutaneous Coronary Intervention CT reconstruction demonstrating the LMS stent following the first procedure, with red lines representing the distance to the left main ostium.

A repeat computed tomography TAVI confirmed the stent had displaced into the left main body from the very oblique left main ostium (Figure 5, Video 5). We elected to implant a 23-mm Sapien S3 with an upfront chimney stent to minimize the risk of coronary occlusion and still provide satisfactory hemodynamic performance in the context of the patient’s overall comorbidity and likely longevity.

**Figure 6:**
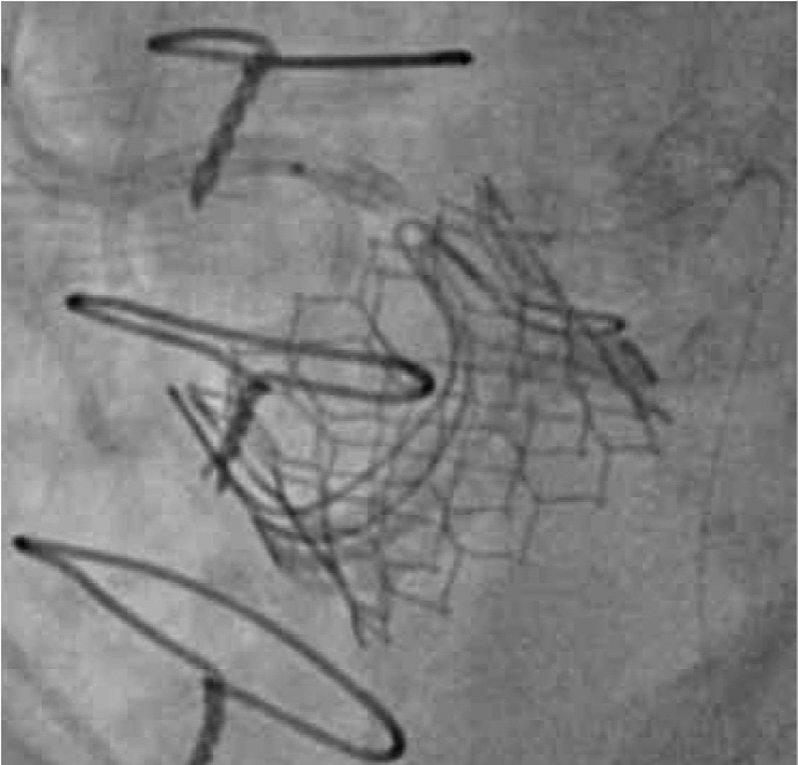
Final Transcatheter Aortic Valve Implantation Image

The TAVI procedure was delayed by approximately 2 months due to lobar pneumonia which required hospitalization. Bifemoral arterial access was obtained under ultrasound guidance. A 7-F AL1 guide and Balance coronary guidewire were used to introduce a 7-F Guidezilla guide extension catheter and a 5.0- × 20-mm Megatron stent. This was positioned and deployed with the proximal end abutting the upper border of the Perimount valve frame at the sinotubular junction and the distal end overlapping the previously deployed left main stent. A 6.0- × 20-mm NC balloon was used to post dilate the stent and was left in situ for the duration of the TAVI (Video 6). The bioprosthetic valve was crossed using an Amplatz left 2 catheter and a straight tipped 0.035-inch guidewire. After rapid ventricular pacing at 200 beats/min, the Sapien S3 valve was deployed in a conventional ViV position. As anticipated, there was lateral displacement and compression of the chimney stent during TAVI valve deployment (Video 6), and it was post dilated with a 6.0 × 20 NC balloon. A pigtail aortogram demonstrated a satisfactory TAVI valve position with no paravalvular leak and a patent left main coronary artery (Figure 6). The invasive peak to peak gradient was 11 mm Hg. The right femoral artery was closed with 2 Perclose sutures and the left femoral artery with a single Perclose suture.

**Visual Summary:**
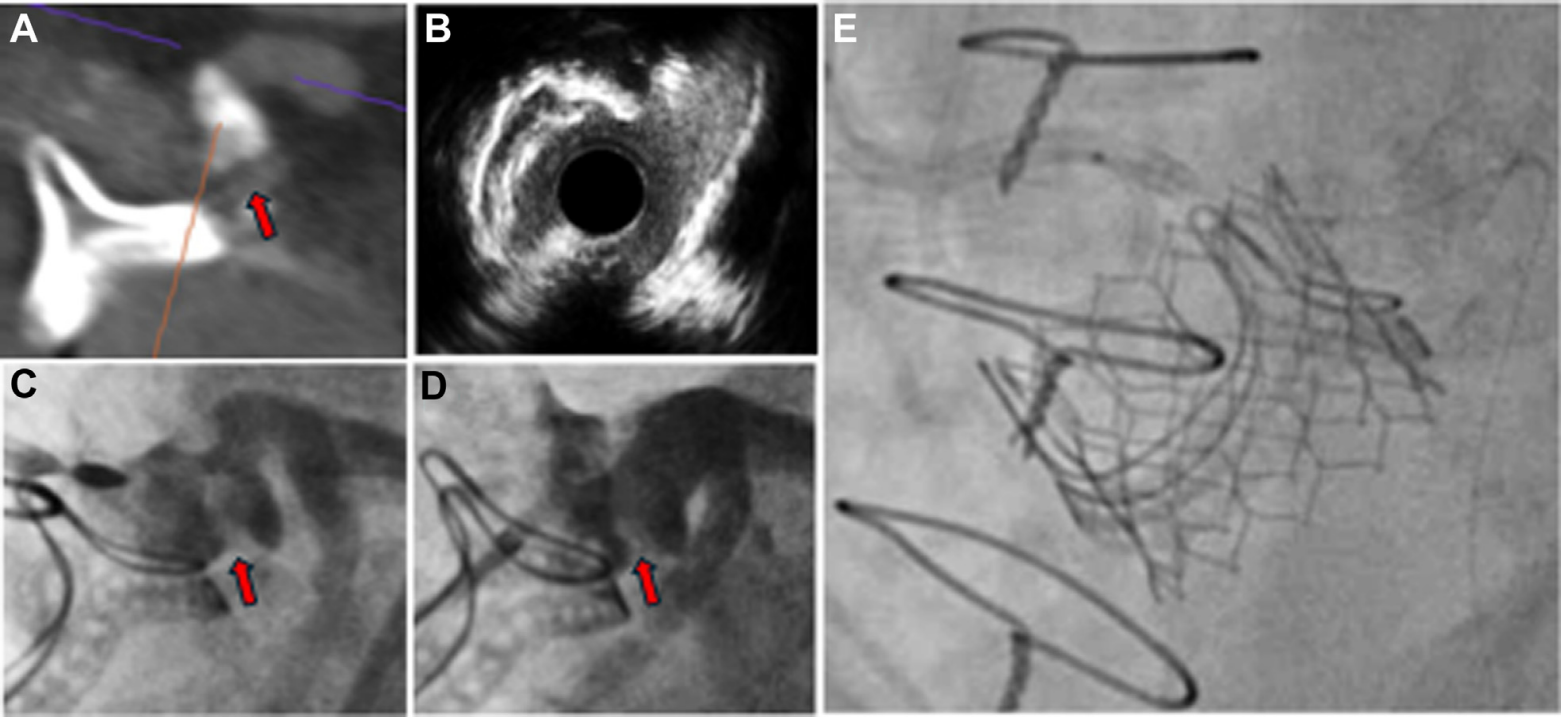
Multi-Modality Imaging Assessment of Calcification Computed tomography reconstruction with dense calcific linear obstruction abutting the left main coronary ostium (A) with evidence of left main body disease and severe left main ostial stenosis on intravascular ultrasound (B). Invasive angiography suggesting a connection with the annular plane in keeping with a retained aortic valve leaflet (C), and evidence of modification after rotablation (D). Final image after valve-in-valve transcatheter aortic valve implantation with a 23-mm Sapien S3 and left main coronary artery chimney stent and disengaged 6.0- × 20-mm noncompliant balloon (E).

A postoperative transthoracic echocardiogram demonstrated a well-seated TAVI valve with no paravalvular leak, a mean gradient of 8 mm Hg, and an aortic valve Vmax of 2.8 m/s. The patient was discharged on day 2.

At 3 months post procedure, the patient remained asymptomatic and well. There were no clinical signs of heart failure. Due to recurrent epistaxis (platelet count: 45), the patient was continued on clopidogrel monotherapy alone.

## Discussion

Concomitant calcific de novo left main coronary disease and severe aortic stenosis is well described and often best served by surgical aortic valve replacement and coronary artery bypass grafting. However, in patients who are not candidates for cardiac surgery or those who require redo sternotomy, rotational atherectomy of de novo left main stem disease to facilitate TAVI has been described. In our case, we observed both aorto-ostial calcification and a linear calcific structure abutting the left main ostium which extended from the annular plane. After multimodality imaging assessment, this was thought to represent a retained aortic valve leaflet. Rotational atherectomy facilitated sufficient debulking to permit left main stenting and ultimately TAVI.

Rotational atherectomy has been successfully used to modify a retained calcified spike which was impeding leaflet motion in a mechanical aortic valve replacement.^1^ In that case, the authors successfully used a Sentinel Cerebral Protection Device. We shared their concern about the potential for stroke when using rotablation on calcific structures within the major central vasculature, and successfully used a similar approach, with no clinical signs of stroke. The other major periprocedural complications we considered were aortic or coronary artery perforation. We minimized this risk through careful guide catheter selection and manipulation.

A 25-mm Perimount valve has a true internal diameter of 23 mm. Typically, a 26-mm Sapien S3 is recommended where the internal bioprosthetic valve diameter is between 21 and 24 mm, to ensure adequate oversizing and achieve satisfactory anchoring.^2^ However, this would have resulted in a VTC of 3.0 mm and increased the risk of annular damage from bulky calcification. Analysis of the Valve-in-Valve International Data registry demonstrates that a shorter VTC distance predicts coronary obstruction, with an optimal cutoff of 4 mm (area under the curve for coronary obstruction: 0.943, *P* < 0.001), and this is associated with a high 30-day mortality (52.9% vs 3.9%).^3^ A 23-mm Sapien S3 increased the VTC distance to 3.9 mm and minimized the risk of annular damage.

The successful outcome in our case was facilitated by multimodality imaging combined with invasive assessment to clarify the mechanism of coronary obstruction, careful multidisciplinary heart team discussion, and procedural planning followed by detailed informed consent.

## Conclusions

Rotational atherectomy was successfully used to modify a retained calcified aortic valve leaflet and aorto-ostial left main calcification. This facilitated left main stem percutaneous coronary intervention and ViV TAVI with a successful outcome. In patients at high risk of coronary obstruction who are not candidates for redo surgery, this approach may be considered after multidisciplinary team discussion, multimodality imaging assessment, and detailed informed consent.

## Funding Support and Author Disclosures

The authors have reported that they have no relationships relevant to the contents of this paper to disclose.

## Appendix

Video 1 Coronary Angiogram Unable to Engage LMS

Video 2 Intravascular Ultrasound of LMS After Repeat Coronary Angiography

Video 3 Intravascular Ultrasound of LMS After Rotational Atherectomy

Video 4 Sentinel Device and LMS Percutaneous Coronary Intervention

Video 5 Intravascular Ultrasound After LMS Percutaneous Coronary Intervention

Video 6 Chimney Stent and Transcatheter Aortic Valve Implantation
